# Does a monetary incentive improve the response to a postal questionnaire in a randomised controlled trial? The MINT incentive study

**DOI:** 10.1186/1745-6215-10-44

**Published:** 2009-06-22

**Authors:** Simon Gates, Mark A Williams, Emma Withers, Esther Williamson, Shahrul Mt-Isa, Sarah E Lamb

**Affiliations:** 1 Warwick Medical School Clinical Trials Unit, University of Warwick, Coventry, UK; 2 Division of Epidemiology, Dept of Epidemiology & Public Health, Imperial College London, St Mary's Campus, Norfolk Place, London, UK

## Abstract

**Background:**

Sending a monetary incentive with postal questionnaires has been found to improve the proportion of responders, in research in non-healthcare settings. However, there is little research on use of incentives to improve follow-up rates in clinical trials, and existing studies are inconclusive. We conducted a randomised trial among participants in the Managing Injuries of the Neck Trial (MINT) to investigate the effects on the proportion of questionnaires returned and overall non-response of sending a £5 gift voucher with a follow-up questionnaire.

**Methods:**

Participants in MINT were randomised to receive either: (a) a £5 gift voucher (incentive group) or (b) no gift voucher (no incentive group), with their 4 month or 8 month follow-up questionnaire. We recorded, for each group, the number of questionnaires returned, the number returned without any chasing from the study office, the overall number of non-responders (after all chasing efforts by the study office), and the costs of following up each group.

**Results:**

2144 participants were randomised, 1070 to the incentive group and 1074 to the no incentive group. The proportion of questionnaires returned (RR 1.10 (95% CI 1.05, 1.16)) and the proportion returned without chasing (RR 1.14 (95% CI 1.05, 1.24) were higher in the incentive group, and the overall non-response rate was lower (RR 0.68 (95% CI 0.53, 0.87)). Adjustment for injury severity and hospital of recruitment to MINT made no difference to these results, and there were no differences in results between the 4-month and 8-month follow up questionnaires. Analysis of costs suggested a cost of £67.29 per additional questionnaire returned.

**Conclusion:**

Monetary incentives may be an effective way to increase the proportion of postal questionnaires returned and minimise loss to follow-up in clinical trials.

**Trial registration number:**

ISRCTN61305297

## Background

Postal questionnaires to participants are used by many randomised controlled trials and other research studies as a method of data collection, especially for long-term follow-up collecting data on quality of life, resource use and patient-reported outcome measures. Failure to return questionnaires results in missing data, which could potentially introduce bias into the trial's results, and strategies are therefore frequently employed to promote questionnaire return. Often this involves a system of follow-up contacts by post and telephone to ensure that data are collected from as many participants as possible. This type of system is labour-intensive, especially if large numbers of questionnaires need to be chased, and other methods of improving questionnaire return rates may also be useful.

The use of a monetary incentive sent with the questionnaire is an easily implemented strategy that may help to increase the proportion returned. A Cochrane systematic review of randomised trials of monetary incentives to promote questionnaire return showed that this intervention was effective (OR 1.99, 95% CI 1.81, 2.18).[1] However, the majority of the studies in this review were not conducted in healthcare settings and none of them involved participants in clinical trials. The relevance of this result to follow-up in clinical trials is therefore questionable. A recent systematic review of the use of incentives in health care settings[2] did not show an improvement in questionnaire return (OR 1.09, 95% CI 0.94, 1.27); however, this analysis included only four studies, and two more recent studies[3,4] have suggested that monetary incentives may have a beneficial effect. There is therefore uncertainty as to whether monetary incentives are effective, and further research is justified.

Use of incentives involves an additional cost (of the incentive itself), but, if the proportion returned is improved, costs involved in following up questionnaires by telephone and post may be saved. Monetary incentives may therefore be able to achieve a useful increase in response at little or no additional cost. Only one study, an observational study conducted alongside a randomised trial in primary care[5], has estimated the costs of using incentives, and it found an additional cost per extra questionnaire returned of £48.28. However, the calculation did not include the costs of staff time, so it is possible that, if using an incentive reduced the effort in chasing non-returned questionnaires, the true additional cost would be lower than this.

Here we report the results of the MINT incentive study, a randomised controlled trial of a £5 incentive, conducted alongside the Managing Injuries of the Neck Trial (MINT).[6] MINT was a cluster randomised trial of advice interventions given in emergency departments to patients presenting with acute whiplash injuries, in which eligible patients were identified by participating emergency departments. Participants were then followed up by postal questionnaires sent from the study office, 4, 8 and 12 months after their injury

## Methods

We included participants in MINT who were being sent a follow-up questionnaire at 4 months or 8 months after their whiplash injury. Each participant was randomised into the incentive study only once; participants who were randomised to receive an incentive or no incentive with their 4 month questionnaire were not randomised again when they reached the 8 month follow-up. We used both follow-up time points to maximise the number of participants that could be included. When the incentive study started, MINT had already been recruiting for about 16 months, and hence randomising participants to incentive or no incentive at only the 4 month time point would have restricted the sample size available. Because the 4 month and 8 month questionnaires were identical, it was reasonable to include both of them in the incentive study, enabling us to randomise participants who had already passed the 4 month follow-up point. The follow-up questionnaires consisted of the Neck Disability Index (NDI)[7], two standard quality of life measures (SF-12 and EQ-5D), and questions on resource use and beliefs about neck pain. The total number of questions was 49. The questionnaire consisted of 15 A4 sized pages, and was sent out with a personalised covering letter and prepaid return envelope. The trial's standard method of chasing non-returned questionnaires was used. This was as follows: a second copy of the questionnaire was sent after two weeks, followed by up to three attempts to make contact by telephone to request return of the questionnaire. Finally, participants were offered the option to provide the most important outcome data by telephone. The questionnaire sent at the 12 month follow-up was slightly different, so this time point was not included in the incentive study.

Participants were randomised to receive either: (a) a £5 gift voucher, redeemable at a range of shops , with their questionnaire, and a covering letter including a sentence explaining that the voucher is to thank participants for their time and effort; or (b) no gift voucher, and a standard covering letter. Allocation to study arms was according to whether a specific digit of the patient's study number was odd or even. Recruitment into the incentive study took place between 26 April 2007 and 15 January 2008. Ethics approval for this study was given by the Trent Multicentre Research Ethics Committee.

We estimated that a sample size of 2,160 (1,080 per arm) would be sufficient to demonstrate an increase in questionnaire return from 53% (the proportion of 4 month follow-up questionnaires returned in the early part of MINT, at the time of planning the incentive study) in the no incentive group, to 60% in the incentive group, at 5% significance level with 90% power (risk ratio of 1.13).

We used risk ratios (RR) and their 95% confidence intervals to compare the proportions of questionnaires returned, the proportion returned without any chasing, and the proportions of participants classified as non-responders after all chasing efforts, between the incentive and no incentive arms. We performed logistic regression analyses to explore the effects of adjusting for potential baseline imbalances in hospital of recruitment and severity of injury. We also performed a subgroup analysis to investigate whether there was any evidence of different effects of incentives at the 4 month and 8 month time points.

We also estimated the cost of following up each group, including the costs of printing questionnaires and envelopes, postage, and staff time to make follow-up telephone calls (using average durations of calls estimated by the trial staff, and 2007 salary data), as well as the costs of the incentives themselves.

## Results

### Baseline characteristics (Table 1)

**Table 1:** Baseline characteristics

	Incentive n = 1070 (%)	No incentive n = 1074 (%)
Centre of recruitment to MINT		
Hospital A	102 (9.5)	104 (9.7)
Hospital B	51 (4.8)	46 (4.3)
Hospital C	156 (14.6)	188 (17.5)
Hospital D	42 (3.9)	36 (3.4)
Hospital E	61 (5.7)	78 (7.3)
Hospital F	54 (5.0)	53 (4.9)
Hospital G	69 (6.4)	64 (6.0)
Hospital H	98 (9.2)	90 (8.4)
Hospital I	62 (5.8)	55 (5.1)
Hospital J	21 (2.0)	22 (2.0)
Hospital K	130 (12.1)	112 (10.4)
Hospital L	53 (5.0)	49 (4.6)
Hospital M	22 (2.1)	23 (2.1)
Hospital N	80 (7.5)	82 (7.6)
Hospital O	69 (6.4)	72 (6.7)
Time point of recruitment		
4 month questionnaire	590 (55.1)	604 (56.2)
8 month questionnaire	480 (44.9)	470 (43.8)
Age, years; mean [sd]	37.0 [13.4]	36.8 [13.2]
range	18–87	18–78
Sex male	448 (41.9)	456 (42.5)
Female	613 (57.3)	606 (56.4)
Missing	9 (0.7)	12 (1.1)
WAD grade at ED assessment		
I	572 (53.5)	617 (57.4)
II	465 (43.5)	429 (39.9)
III	33 (3.1)	28 (2.6)

**Figure 1:**
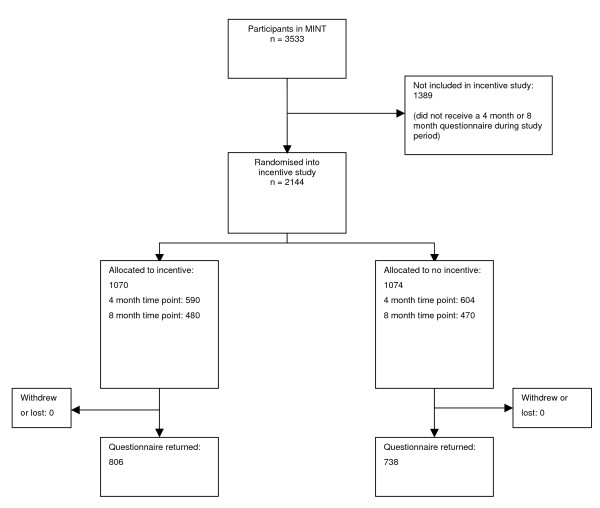
**CONSORT diagram**.

The total number of participants in the incentive study was 2,144 (Figure 1). Of these, 1,194 were randomised at the 4 month time point, and 950 at 8 months. Balance between the study arms in patient characteristics and centre of recruitment to MINT was good. There were only small imbalances in centre of recruitment and in severity of injury, measured by the WAD (Whiplash Associated Disorder) grading system[8].

### Effects of incentives (Table 2)

**Table 2:** Outcomes

	Incentive n = 1070 (%)	No incentive n = 1074 (%)	Risk ratio
Questionnaire returned	810 (75.7)	738 (68.7)	1.10 (1.05, 1.16)
Questionnaire returned without chasing	560 (52.3)	493 (45.9)	1.14 (1.05, 1.24)
Non-responder (after all follow-up efforts including outcome data collection by telephone)	96 (9.0)	142 (13.2)	0.68 (0.53, 0.87)

People who received an incentive were more likely to return the questionnaire (RR 1.10 (95% confidence interval (CI) 1.05, 1.16)), more likely to return the questionnaire without the need for any chasing by the study office (i.e. sending a second copy of the questionnaire and subsequent phone calls) (RR 1.14 (95% CI 1.05, 1.24), and less likely to be a non-responder after all data-chasing efforts (including collection of core outcomes by telephone for people who did not return the questionnaire) (RR 0.68 (95% CI 0.53, 0.87)). The risk difference for return of the questionnaire was +0.07 (95% CI +0.03, +0.11); this corresponds to 14 incentives being sent for one additional questionnaire returned. There was no difference in the time taken to return questionnaires (mean difference = -0.55 days (95% CI -2.27, 1.17). The subgroup analysis showed no evidence of different effects at the 4 month and 8 month time points (Table 3). Among participants randomised at the 4-month time point, there was a small improvement in the proportion of 8 month questionnaires returned in the incentive group (incentive group 511/590, no incentive group 492/604, RR 1.06 (95% CI 1.01, 1.12).

**Table 3:** Subgroup analysis

	4 month questionnaire		8 month questionnaire		
	Incentive n = 590 (%)	No incentive n = 604 (%)	Incentive n = 480 (%)	No incentive n = 470 (%)	Interaction stats Ratio of RR, 4 months versus 8 months (95% CI)
Questionnaire returned	473 (80.2)	435 (72.0)	337(70.2)	303 (64.5)	1.02 (0.92, 1.14)
Questionnaire returned without chasing	338 (57.3)	287 (47.5)	222 (46.3)	206 (43.8)	1.14 (0.96, 1.37)
Non-responder (after all follow-up efforts)	46 (7.8)	73 (12.1)	50 (10.4)	69 (14.7)	0.91 (0.56, 1.48)

Logistic regression controlling for hospital of recruitment to MINT and WAD grade showed that the results for questionnaire return were unaffected. The unadjusted odds ratio for incentive versus no incentive was 1.43 (95% CI 1.17, 1.72); adjustment for hospital of recruitment had a very small effect (χ^2 ^= 20.31, 14 df, p = 0.12, adjusted OR 1.42 (95% CI 1.18, 1.72)), as did adjustment for WAD grade (χ^2 ^= 2.45, 2 df, p = 0.29, adjusted OR 1.41 (95% CI 1.17, 1.72)).

The analysis of costs (Table 4) showed an average cost per participant of £9.35 in the incentive group and £4.64 in the no incentive group. Costs per questionnaire returned were £12.35 in the incentive group and £6.76 in the no incentive group. The cost per additional questionnaire returned was £67.29 and the cost per additional overall response (including data collection by telephone) was £110.62.

**Table 4:** Costs

		Number	
	Cost	Incentive	No incentive
Randomised		1070	1074
Initial mailing (printing, envelope, postage)	£1.25	1070	1074
Incentive	£5	1070	0
Repeat copy of mailing (printing, envelope, postage)	£1.25	504	577
First follow-up phone call	£0.90	808	844
Second follow-up phone call	£0.90	497	578
Telephone data collection – unsuccessful	£1.09	396	472
Telephone data collection – successful	£6.54	101	115
Questionnaire returned	£0.52	810	738
			
Cost per participant randomised		£9.35	£4.64
Cost per questionnaire returned		£12.35	£6.76

## Discussion

In this trial, sending an incentive resulted in an increase in the proportion of questionnaires returned and a decrease in the number of non-responders. This indicates that incentives may be able to give a useful improvement in questionnaire return and help to reduce the quantity of missing data in trials, and hence improve the quality of evidence they provide. In the population studied here, sending an incentive produced a moderate improvement in questionnaire return for minimal extra effort on the part of the trial staff. The size of the increase in the proportion returned (an absolute difference of 7%) was similar to that found by two other recent studies. Dirmaier et al[3], in a study of patients being followed up after inpatient treatment for mental health problems, found a 7.3% increase with a small monetary incentive (with a value of 5 Deutschmarks), and Kenyon et al[4] found an improvement of 11.7% with a £5 gift voucher, in a study of long-term follow-up of participants in a randomised trial of antibiotics for preterm labour[9,10].

The main cost involved in using incentives is the additional cost of the incentives themselves. These costs were not offset by a corresponding reduction in the costs of chasing non-returned questionnaires; the difference in cost per participant between the incentive and no incentive groups was close to the cost of the incentive. The cost per additional questionnaire returned was about £67, which is comparable to the cost estimated by Brealey et al[5]. In a trial of 1000 patients, therefore, the additional cost to improve the proportion of questionnaires returned by 7% would be less than £5,000. This is a small amount in the context of current trial budgets, where the cost per recruit often exceeds £1,000, and provision of an unconditional incentive is therefore a feasible strategy.

We used a quasi-randomisation procedure rather than true randomisation to allocate participants to the incentive and non-incentive arms. This was adopted because of the practical difficulty of performing a true randomisation at the time questionnaires were sent out. The study numbers were allocated consecutively in the hospital emergency departments at the time that that potential participants were first identified. Not all of these patients agreed to join MINT, hence the trial population did not contain exactly equal numbers of participants with odd and even digits. It is extremely unlikely that there was any association between odd and even digits and any patient characteristics, and therefore there were unlikely to have been any systematic differences between the incentive and no-incentive groups. The lack of concealment of allocations before randomisation was not a major concern because it would have been very difficult for the staff in the study office who were sending out the questionnaires to have selectively allocated systematically different patients to the trial arms. Similarly, although the trial office staff were unblinded, this was very unlikely to have introduced bias, as they had no influence over any participant's decision to return the questionnaire, and postal and telephone follow-up contacts were performed in a standardised way for all participants, without any reference to whether or not they were participating in the incentive study.

In this study we regarded vouchers as a monetary incentive, but it is not known if they are valued in the same way as cash, or whether the effects of a cash incentive would be similar. It seems likely that this type of voucher, which can be exchanged for goods in a large number of shops, in the same way as cash, would be valued by recipients similarly to cash. Several trials of non-monetary incentive were included in the Cochrane review[1], and were analysed separately from monetary incentives. These non-monetary incentives included small gifts such as pens, lottery tickets, entry to a prize draw, or donations to charity. Our view is that the vouchers evaluated in this trial would be valued much more similarly to cash than to these types of non-monetary incentive.

Several questions remain about the use of monetary incentives to promote questionnaire return. It is not yet clear whether (and how) the effectiveness of incentives varies between different populations and different types of trial. Incentives have not yet been tested in a wide enough variety of populations to be confident of their general applicability. It is possible that they may be most useful in situations where proportions returned are often low, for example in trials of acute injuries, where most participants will have recovered by the time of follow-up and may have little motivation to return questionnaires. The most cost-effective size of incentive is uncertain; for convenience we followed an earlier study in using a £5 gift voucher, but it is not known whether a smaller incentive would have been equally effective, or a larger one more effective. Existing evidence, from a systematic review of trials mainly in non-patient populations, suggests that there is a non-linear relationship between the size of the incentive and the improvement in response.[11] In this review there was an increase in the odds ratio for response up to an incentive size of $5 (US), but no further increase beyond this.

This study adds to the evidence that incentives may be an effective means of reducing the amount of missing data with postal data collection in clinical trials. Questions remain about how they might best be used. In this study we used an "unconditional" incentive, that was sent out with the questionnaire, rather than a "conditional" incentive, provided only once a questionnaire is returned[1]. Existing evidence suggests that unconditional incentives are more effective, and they are also administratively simpler. However, almost all of this evidence is not from participants in health research studies, and its applicability to these populations is unknown. Furthermore, in many research studies (including MINT), participants are followed up at several time points, and the aim is to sustain a high rate of questionnaire return over a year or more. The most cost-effective use of incentives in this situation is uncertain. It may be most effective, but also most costly, to send an incentive with every questionnaire, but there is a possibility that effectiveness might decline with repetition, resulting in a lower proportion returned for later questionnaires. If the primary outcome is measured at one particular time point, an incentive could be used for this questionnaire only, but this may risk a poor response at earlier time points. Alternatively, it may be best to send an incentive with the first questionnaire, as its effects may carry over to subsequent time points. Our results in this study suggest that there may be some "carry over" effect, as receipt of an incentive with the 4 month questionnaire improved the proportion of 8 month questionnaires returned.

## Conclusion

A small financial incentive in the form of a £5 gift voucher gave an absolute increase in the proportion of questionnaires returned of about 7% and a relative increase of 10%. The cost per additional questionnaire returned was low.

## Competing interests

The authors declare that they have no competing interests.

## Authors' contributions

SG, SEL, MAW and EmW were responsible for the study conception and design. MAW, EmW, EsW and SMI were responsible for the study conduct. EmW, SMI and MAW were responsible for data management. SG was responsible for analysis and drafting the manuscript. All authors were responsible for revising the manuscript.

## References

[B1] Edwards PJ, Roberts IG, Clarke MJ, DiGuiseppi C, Wentz R, Kwan I, Cooper R, Felix L, Pratap S (2007). Methods to increase response rates to postal questionnaires. Cochrane Database Syst Rev.

[B2] Nakash RA, Hutton JL, Jorstad-Stein EC, Gates S, Lamb SE, Nakash RA, Hutton JL, Jorstad-Stein EC, Gates S, Lamb SE (2006). Maximising response to postal questionnaires-a systematic review of randomised trials in health research. BMC Med Res Methodol.

[B3] Dirmaier J, Harfst T, Koch U, Schulz H, Dirmaier J, Harfst T, Koch U, Schulz H (2007). Incentives increased return rates but did not influence partial nonresponse or treatment outcome in a randomized trial. J Clin Epidemiol.

[B4] Kenyon S, Pike K, Jones D, Taylor D, Salt A, Marlow N, Brocklehurst P, Kenyon S, Pike K, Jones D (2005). The effect of a monetary incentive on return of a postal health and development questionnaire: a randomised trial [ISRCTN53994660]. BMC Health Serv Res.

[B5] Brealey SD, Atwell C, Bryan S, Coulton S, Cox H, Cross B, Fylan F, Garratt A, Gilbert FJ, Gillan MG (2007). Improving response rates using a monetary incentive for patient completion of questionnaires: an observational study. BMC Med Res Methodol.

[B6] Lamb SE, Gates S, Underwood MR, Cooke MW, Ashby D, Szczepura A, Williams MA, Williamson EM, Withers EJ, Mt-Isa S (2007). Managing Injuries of the Neck Trial (MINT): design of a randomised controlled trial of treatments for whiplash associated disorders. BMC Musculoskelet Disord.

[B7] Vernon H, Mior S (1991). The Neck Disability Index: a study of reliability and validity. J Manipulative Physiol Ther.

[B8] Spitzer WO, Skovron ML, Salmi LR, Cassidy JD, Duranceau J, Suissa S, Zeiss E (1995). Scientific monograph of the Quebec Task Force on Whiplash-Associated Disorders: redefining "whiplash" and its management. Spine.

[B9] Kenyon SL, Taylor DJ, Tarnow-Mordi W, Group OC (2001). Broad-spectrum antibiotics for preterm, prelabour rupture of fetal membranes: the ORACLE I randomised trial. ORACLE Collaborative Group. Lancet.

[B10] Kenyon SL, Taylor DJ, Tarnow-Mordi W, Group OC (2001). Broad-spectrum antibiotics for spontaneous preterm labour: the ORACLE II randomised trial. ORACLE Collaborative Group. Lancet.

[B11] Edwards P, Cooper R, Roberts I, Frost C (2005). Meta-analysis of randomised trials of monetary incentives and response to mailed questionnaires. J Epidemiol Community Health.

